# Mental disorders due to Covid-19 in the Turkish population

**DOI:** 10.1192/j.eurpsy.2022.859

**Published:** 2022-09-01

**Authors:** B. Mykhaylov, O. Kudinova, E. Kavak

**Affiliations:** 1 Private Higher Education Establishment “Kyiv medical university”, Psychiatry, Kyiv, Ukraine; 2 Kharkiv Medical Academy of Postgraduate Education, Psychotherapy, Kharkiv, Ukraine

**Keywords:** Covid-19, Mental Disorders, Anxiety

## Abstract

**Introduction:**

The COVID-19 pandemic grabs attention to the study and solution of this global problem around the world.

**Objectives:**

The aim of the study is to link the level of anxiety and fear that emerged in Turkey during the epidemic.

**Methods:**

A survey was conducted, which contains 10 questions. 433 people took part, 5- 15 - 18 (1.2%), 168 - 19 - 29 years (38.8%), 202 - 30 - 39 (46.7%), 47 - 40-49 (10, 9%) 11 from 50 years (2.5%).

**Results:**

Men with panic attack criteria - 11.3% (11 people), women - 9.8% (35 people). Symptoms of GTR were found in 31% (25) men and 18% (66) women. 328 participants - university graduates: 29% believe that the information of the Ministry of Health is correct. No significant difference between the percentages of compliance with the criteria for diagnosing panic attacks and anxiety was found depending on educational status. 8.3% of participants, 36 people, were diagnosed with Covid. The share of those who did not have a code, but thought they had, is 42%, and among those who had a code (before the disease) - 72%. There is a direct relationship between the level of education and personal protection against epidemics.

**Conclusions:**

From a community mental health perspective, it is important that all covid-positive patients receive psychiatric support, whether or not they meet the DSM-V and ISD 10 diagnostic criteria.

**Disclosure:**

No significant relationships.

